# High Precision Compensation for a Total Reflection Prism Laser Gyro Bias in Consideration of High Frequency Oscillator Voltage

**DOI:** 10.3390/s19132986

**Published:** 2019-07-06

**Authors:** Yuanbo Tao, Sihai Li, Jiangtao Zheng, Feng Wu, Qiangwen Fu

**Affiliations:** 1 School of Automation, Northwestern Polytechnical University, Xi’an 710072, China; 2 Shanghai Aerospace Control Technology Institute, Shanghai 201109, China

**Keywords:** total reflection prism laser gyro (TRPLG), complex TRPLG bias variation, high frequency oscillator voltage (UHFO), iterative re-weighted least squares support vector machine (IR-LSSVM), TRPLG bias stability

## Abstract

Traditional compensation methods based on temperature-related parameters are not effective for complex total reflection prism laser gyro (TRPLG) bias variation. Because the high frequency oscillator voltage (UHFO) fundamentally affects the TRPLG bias, and the UHFO has a stronger correlation with the TRPLG bias when compared with the temperature, an introduction of UHFO into the TRPLG bias compensation can be evaluated. In consideration of the limitations of least squares (LS) regression and multivariate stepwise regression, we proposed a compensation method for TRPLG bias based on iterative re-weighted least squares support vector machine (IR-LSSVM) and compared with LS regression, stepwise regression, and LSSVM algorithm in large temperature cycling experiments. When temperature, slope of temperature variation, and UHFO were selected as inputs, the IR-LSSVM based on myriad weight function improved the TRPLG bias stability by 61.19% to reach the maximum and eliminated TRPLG bias drift. In addition, the UHFO proved to be the most important parameter in the process of TRPLG bias compensation; accordingly, it can alleviate the shortcomings of traditional compensation based on temperature-related parameters and can greatly improve the TRPLG bias stability.

## 1. Introduction

The total reflection prism laser gyros (TRPLGs) are applied in several systems [1]. For example, the I42-1-C strapdown navigation system of civilian aircrafts IL-96-300 and TU-204 is based on TRPLG. The development of total reflection prism cavities with high Q-factor, small polarization non-reciprocity, and low back-scattering increases TRPLG accuracy and opens new applications such as precise measuring systems for railways as well as oil and gas pipe lines [2].

There are three major errors in conventional two-mode active laser gyros: mode locking, scale factor error, and null shift [3,4]. Mode locking is caused by a weak coupling mechanism between two otherwise independent traveling waves that causes backscattering from one wave into the other, which occurs mostly at the mirrors due to surface imperfections [5,6,7]. Some compensation methods have been developed to rectify this error [8,9,10,11]. Scale factor error refers to variations in the scale factor as a function of the rotation rate [12,13]. Null shift happens when the frequency difference is non-zero for zero input rate and is the most significant error pertaining to accuracy. Because the bias of ring laser gyro (RLG) is sensitive to temperature variations [14,15], it is important to establish an accurate RLG bias temperature model to reduce the effect of the latter [16].

At present, the traditional temperature compensation method of RLG bias is LS regression [17], which is subject to certain restrictions due to complex nonlinear relationships. Second-order multivariate stepwise regression model, which is based on temperature, slope of temperature variation, and its cross terms, can improve partial compensation effect [18]. The neural network technology, a parallel distributed system which overcomes the defects of traditional logic symbol-based artificial intelligence in dealing with intuitive and unstructured information, has seen rapid advances in recent years. It has the characteristics of self-adaptation, self-organization, and real-time learning [19]. However, due to empirical risk minimization, it is easy to achieve local rather than global optimization with this method. Therefore, Vapnik et al. developed a support vector machine (SVM) that has the characteristics of structural risk minimization [20]. The training of SVM is a quadratic optimization problem with a unique solution, making it optimal on a global scale. Moreover, SVM has a better generalization ability than those offered by neural networks. On account of the above advantages, SVM has been widely used in the fields of classification, regression, pattern recognition, and image processing [21]. However, the SVM algorithm is difficult to implement for large-scale training samples because the storage and calculation of the matrix requires considerable machine memory and computation time. Therefore, Suykens et al. proposed the least squares support vector machine (LSSVM), which starts from the loss function and converts the inequality constraint into an equality constraint, thus transforming the optimization problem into a solving linear equation problem [22]. Finally, the computational complexity of the SVM is avoided and high modeling accuracy is preserved. The LSSVM has been widely applied to the temperature compensation of the RLG bias [23]. However, a single temperature compensation method does not have much effect on complex RLG bias changes. Therefore, other output signals for RLG bias compensation are being studied [24]. However, LSSVM has two main disadvantages, it is sensitive to outliers and lacks sparseness, which limits the method when training large-scale problems. To overcome the former disadvantage, Suykens et al. proposed a weighted LSSVM (W-LSSVM) algorithm [25] and Bao et al. proposed an iterative re-weighted LSSVM (IR-LSSVM) algorithm [26,27]. In addition, some techniques utilize non-convex loss functions to improve robustness [28,29,30,31], which is known as robust LSSVM (R-LSSVM). To overcome the latter disadvantage, Chen et al. proposed a sparse R-LSSVM (SR-LSSVM) to achieve a sparse solution of the primal R-LSSVM after obtaining a low-rank approximation of the kernel matrix [32]. Recently, these LSSVM-based improved algorithms have not been applied to the compensation of RLG bias.

The TRPLG differs from the traditional RLG in the structure and control principle of the resonant cavity [33,34]. Therefore, the factors causing the gyro bias are not exactly the same. In view of the particularity of the TRPLG output, this paper does not use the latest regression algorithm and adopts the more suitable IR-LSSVM algorithm. Further, the UHFO is also considered. This paper examines high precision compensation for TRPLG bias in consideration of the UHFO. In Section 2, the TRPLG bias compensation model, the LSSVM and IR-LSSVM algorithm are described. Experimental configuration is shown in Section 3. In Section 4, large temperature cycling experiments verify the importance of the UHFO and the effectiveness of the proposed method. Finally, Section 5 contains the conclusions of the study.

## 2. Model and Algorithm of TRPLG Bias Compensation

### 2.1. TRPLG Parameters Used for Bias Compensation

**Figure 1:**
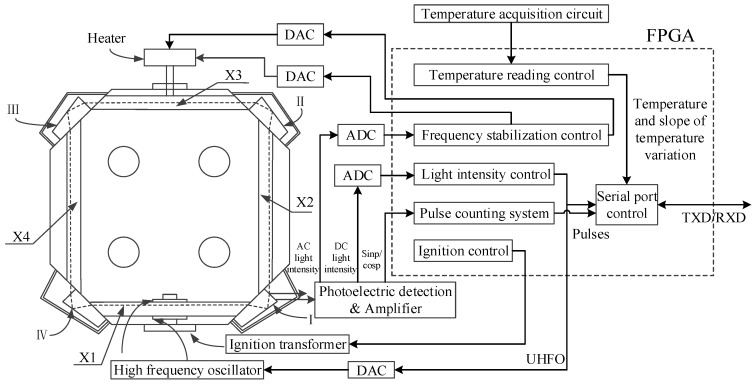
Basic block diagram of TRPLG (total reflection prism laser gyro).

Basic block diagram of TRPLG is shown in Figure 1. The core component was a resonant microcrystalline glass cavity with fused silica glass prisms [2]. The X1 channel was filled with helium neon gas mixed in a certain proportion with a working wavelength of 0.6328 µm. The laser was generated under the action of ignition transformer and high frequency oscillator. X2 and X4 were vacuum channels, and the X3 channel was filled with dry air. The refractive index of the air was changed by connecting the heater to stabilize the resonant frequency of the cavity. The parts marked as I, II, III, and IV were prisms, which were sealed by a protective cover to keep their surfaces clean. The control circuit consisted of field programmable gate array (FPGA) and other peripheral circuits, which mainly include photoelectric detection and amplification, analog to digital converter (ADC), digital to analog converter (DAC), high frequency oscillator, ignition transformer, and temperature acquisition. The major functions completed by the FPGA included ignition control, light intensity control, frequency stabilization control, temperature reading control, pulse counting system, and serial port control. The ignition control system provided the ignition pulse signal for ignition transformer, and the light intensity control system generated UHFO according to the DC light intensity to maintain its stability. The frequency stabilization control system generated the heater voltage based on AC light intensity to maintain the stability of the resonator frequency. The temperature reading control completed reading the temperature, and the pulse counting system was for phase detection, high-speed sampling, and low-pass filtering. Finally, useful information was read through the serial port control system.

**Figure 2:**
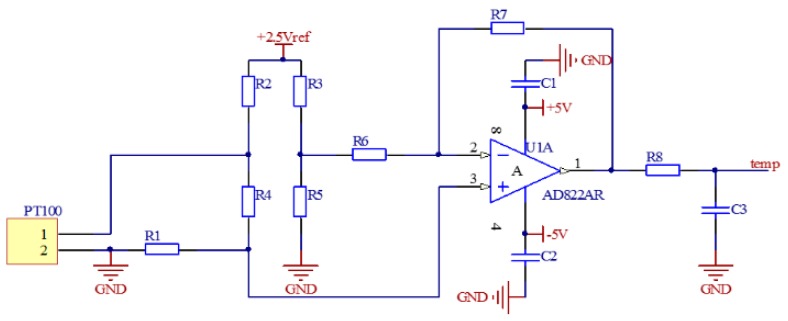
Temperature acquisition circuit.

**Figure 3:**
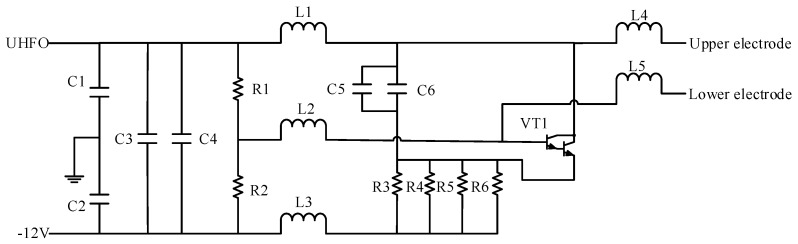
High frequency oscillator circuit.

The main factors that affect TRPLG bias are temperature, slope of temperature variation, and UHFO. The temperature variation causes a change in the refraction index of the prism, which causes a change in the optical path and its length, eventually altering the TRPLG bias. The temperature acquisition circuit is shown in Figure 2. PT100 was used to convert the temperature into a resistance, which converts the voltage signal through this circuit. Therefore, FPGA controls ADC to acquire it. The temperature acquisition circuit consists of a bridge circuit, an operational amplifier, and a low-pass filter. The high frequency oscillator circuit is depicted in Figure 3. UHFO served as the input to the circuit while the output was a low-voltage AC signal of 150MHz, which acts on the upper and lower electrodes of the X1 channel. Therefore, the UHFO affected the size of the output voltage as well as the He-Ne gas excitation and TRPLG bias.

**Figure 4:**
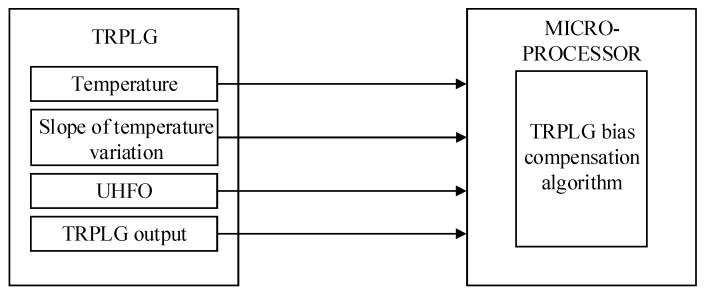
The hardware structure of TRPLG bias compensation.

The hardware structure of TRPLG bias compensation is shown in Figure 4. Temperature, slope of temperature variation, UHFO, and TRPLG output were inputs to the microprocessor through the serial port, and the micro-processor compensated for the TRPLG bias using the TRPLG bias compensation algorithm. In this study, IR-LSSVM was used as the high precision compensation algorithm and was compared with the LS, stepwise regression, and LSSVM. In addition, the influence of UHFO on TRPLG bias compensation was the focus of study.

### 2.2. LSSVM for Nonlinear Function Regression

In the original weight space, the model is calculated as follows

(1) $$y\left(x\right)=\omega^{T}\varphi \left(x\right)+b$$

with given training data ${\left\{x_{k},y_{k}\right\}}_{k=1}^{N}$ and φ(⋅):${\mathbb{R}}^{n}\to {\mathbb{R}}^{n_{h}}$ mapped into high dimensional feature space, it can be of infinite dimension but implicitly defined. The $\omega^{T}$ and b are two unknown variables. In this nonlinear case, the vector ω can also become infinite dimensional. For standard SVM, the optimization problem in the original weight space becomes [21]:

(2) $$\begin{array}{l} \underset{\omega ,b,\xi ,\xi^{*}}{\min}J_{P}\left(\omega ,\xi ,\xi^{*}\right)=\frac{1}{2}\omega^{T}\omega +c\sum_{k=1}^{N}\left(\xi_{k}+\xi_{k}^{*}\right)\\ \;\mathrm{subject}\;\mathrm{to}\;\left\{ \begin{array}{l} y_{k}-\omega^{T}\varphi \left(x_{k}\right)-b\leq \varepsilon +\xi_{k}\;,\;k=1,\ldots ,N\\ \omega^{T}\varphi \left(x_{k}\right)+b-y_{k}\leq \varepsilon +\xi_{k}^{*}\;,\;k=1,\ldots ,N\\ \xi_{k},\xi_{k}^{*}\geq 0\;,\;k=1,\ldots ,N \end{array}\right. \end{array}$$

where $\xi_{k}^{\left(*\right)}$ represents the upper (lower) training error at data point $\left(x_{k},y_{k}\right)$, c represents penalty factor, and ε represents the coefficient of regression estimation accuracy.

After using Lagrangian and optimal conditions, the following double problems are obtained:

(3) $$\begin{array}{l} \underset{\alpha ,\alpha^{*}}{\max}J_{D}\left(\alpha ,\alpha^{*}\right)=-\frac{1}{2}\sum_{k,\;l=1}^{N}\left(\alpha_{k}-\alpha_{k}^{*}\right)\left(\alpha_{l}-\alpha_{l}^{*}\right)K\left(x_{k},x_{l}\right)\\ \;\;\;\;\;\;\;-\varepsilon \sum_{k=1}^{N}\left(\alpha_{k}+\alpha_{k}^{*}\right)+\sum_{k=1}^{N}y_{k}\left(\alpha_{k}-\alpha_{k}^{*}\right)\\ \mathrm{subject}\;\mathrm{to}\;\left\{ \begin{array}{l} \sum_{k=1}^{N}\left(\alpha_{k}-\alpha_{k}^{*}\right)=0\\ \alpha_{k},\alpha_{k}^{*}\in \left[0,c\right] \end{array}\right. \end{array}$$

Core skills have been applied here $K\left(x_{k},x_{l}\right)=\varphi {\left(x_{k}\right)}^{T}\varphi \left(x_{l}\right)$ for k,l=1,…,N. The double representation of the model becomes:

(4) $$y\left(x\right)=\sum_{k=1}^{N}\left(\alpha_{k}-\alpha_{k}^{*}\right)K\left(x,x_{k}\right)+b$$

where $\alpha_{k}$, $\alpha_{k}^{*}$ are the solution to the quadratic programming (QP) problem. The bias term b follows from the complementarity Karush Kuhn Tucker (KKT) conditions. The solution to the QP problem is global and unique provided that the chosen kernel function is positive definite.

With standard SVM as above in the solution of the QP problem, the number of training samples has a significant influence on the matrix scale, resulting in an excessive calculation amount and low calculation speed. Therefore, the extended algorithm LSSVM is studied. The equality constraints are used instead of the inequality constraints. Therefore, this optimization problem described in Equation (2) becomes as follows:

(5) $$\begin{array}{l} \underset{\omega ,b,\xi }{\min}J_{P}\left(\omega ,\xi \right)=\frac{1}{2}\omega^{T}\omega +c\frac{1}{2}\sum_{k=1}^{N}\xi_{k}^{2}\\ \mathrm{subject}\;\mathrm{to}\;y_{k}=\omega^{T}\varphi \left(x_{k}\right)+b+\xi_{k},\;k=1,\ldots ,N. \end{array}$$

This is just a ridge regression cost function formulated in the feature space. However, it should be noted that, when ω becomes an infinite dimension, the original problem cannot be solved. Therefore, we continued building Lagrange and exporting double questions.

One constructs the Lagrangian as:

(6) $$L\left(\omega ,b,\xi ;\alpha \right)=J_{P}\left(\omega ,\xi \right)-\sum_{k=1}^{N}\alpha_{k}\left\{\omega^{T}\varphi \left(x_{k}\right)+b+\xi_{k}-y_{k}\right\}$$

where $\alpha_{k}$ are Lagrange multipliers. The conditions for optimality are given by:

(7) $$\left\{ \begin{array}{l} \frac{\partial L}{\partial \omega }=0\to \omega =\sum_{k=1}^{N}\alpha_{k}\varphi \left(x_{k}\right)\\ \frac{\partial L}{\partial b}=0\to \sum_{k=1}^{N}\alpha_{k}=0\\ \frac{\partial L}{\partial \xi_{k}}=0\to \alpha_{k}=c\xi_{k},\;\;\;\;\;\;\;k=1,\ldots ,N\\ \frac{\partial L}{\partial \alpha_{k}}=0\to \omega^{T}\varphi \left(x_{k}\right)+b+\xi_{k}-y_{k}=0,\;k=1,\ldots ,N. \end{array}\right.$$

After elimination of the variables ω and ξ one gets the following solution:

(8) $$\left[\begin{array}{cccc} 0 & 1 & \cdots & 1\\ 1 & K\left(x_{1},x_{1}\right)+1/c & \cdots & K\left(x_{1},x_{N}\right)\\ \vdots & \vdots & \ddots & \vdots \\ 1 & K\left(x_{N},x_{1}\right) & \cdots & K\left(x_{N},x_{N}\right)+1/c \end{array}\right]\;\left[\begin{array}{c} b\\ \alpha_{1}\\ \vdots \\ \alpha_{N} \end{array}\right]=\left[\begin{array}{c} 0\\ y_{1}\\ \vdots \\ y_{N} \end{array}\right]$$

The core skills of applying here are as follows:

(9) $$K\left(x_{k},x_{l}\right)=\varphi {\left(x_{k}\right)}^{T}\varphi \left(x_{l}\right)k,l=1,\ldots ,N.$$

The resulting LSSVM model for function estimation becomes then [21]

(10) $$y\left(x\right)=\sum_{k=1}^{N}\alpha_{k}K\left(x,x_{k}\right)+b$$

In accordance with Mercer’s condition, many kinds of kernel function $K\left(x,x_{k}\right)$ satisfy $K\left(x_{k},x_{l}\right)=\varphi {\left(x_{k}\right)}^{T}\varphi \left(x_{l}\right)$. In this study, we used a Gaussian function as the kernel function as follows:

(11) $$K\left(x,x_{k}\right)=\exp\left(-{\left\|x-x_{k}\right\|}^{2}/2\sigma^{2}\right)$$

where σ represents the Kernel width. The above regression problem has only two additional tuning parameters (c, $\sigma^{2}$) in LSSVM. After the quadratic optimization is transformed into the solution of the linear equations, the computational efficiency is greatly improved.

### 2.3. Regression by IR-LSSVM

To obtain a robust regression based on the LSSVM solution, one can weight the error variables $\xi_{k}=\alpha_{k}/c$ by weighting factors $\upsilon_{k}$. This leads to the optimization problem [25]:

(12) $$\begin{array}{l} \underset{\omega^{*},b^{*},\xi^{*}}{\min}J_{P}\left(\omega^{*},\xi^{*}\right)=\frac{1}{2}\omega^{*}{}^{T}\omega^{*}+c\frac{1}{2}\sum_{k=1}^{N}\upsilon_{k}\xi_{k}^{*2}\\ \mathrm{subject}\;\mathrm{to}\;y_{k}=\omega^{*}{}^{T}\varphi \left(x_{k}\right)+b^{*}+\xi_{k}^{*},\;k=1,\ldots ,N. \end{array}$$

The Lagrangian becomes:

(13) $$L\left(\omega^{*},b^{*},\xi^{*};\alpha^{*}\right)=J_{P}\left(\omega^{*},\xi^{*}\right)-\sum_{k=1}^{N}\alpha_{k}^{*}\left\{\omega^{*T}\varphi \left(x_{k}\right)+b^{*}+\xi_{k}^{*}-y_{k}\right\}$$

The unknown variables for this weighted LSSVM problem are denoted by the ∗ symbol. From the conditions for optimality and regression of $\omega^{*},\xi^{*}$ one obtains the KKT system

(14) $$\left[\begin{array}{cccc} 0 & 1 & \cdots & 1\\ 1 & K\left(x_{1},x_{1}\right)+1/\left(c\upsilon_{1}\right) & \cdots & K\left(x_{1},x_{N}\right)\\ \vdots & \vdots & \ddots & \vdots \\ 1 & K\left(x_{N},x_{1}\right) & \cdots & K\left(x_{N},x_{N}\right)+1/\left(c\upsilon_{N}\right) \end{array}\right]\;\left[\begin{array}{c} b^{*}\\ \alpha_{1}^{*}\\ \vdots \\ \alpha_{N}^{*} \end{array}\right]=\left[\begin{array}{c} 0\\ y_{1}\\ \vdots \\ y_{N} \end{array}\right]$$

The choice of the weights $\upsilon_{k}$ is determined based on the error variables $\xi_{k}=\alpha_{k}/c$ from the (unweighted) LSSVM case (8). Robust regression is obtained by the Huber, Hampel, Logistic, and Myriad weight functions.

Huber weight function is

(15) $$\upsilon_{k}=\{\begin{array}{cc} 1 & \mathrm{if}\;\left|\xi_{k}/\hat{s}\right|<\beta \\ \frac{\beta }{\left|\xi_{k}/\hat{s}\right|} & \mathrm{if}\;\left|\xi_{k}/\hat{s}\right|\geq \beta \end{array}$$

Hampel weight function is

(16) $$\upsilon_{k}=\{\begin{array}{cc} 1 & \mathrm{if}\;\left|\xi_{k}/\hat{s}\right|<b_{1}\\ \frac{b_{2}-\left|\xi_{k}/\hat{s}\right|}{b_{2}-b_{1}} & \mathrm{if}\;b_{1}\leq \left|\xi_{k}/\hat{s}\right|\leq b_{2}\\ 0 & \mathrm{if}\;\left|\xi_{k}/\hat{s}\right|>b_{2} \end{array}$$

Logistic weight function is

(17) $$\upsilon_{k}=\frac{\tanh\left(\xi_{k}/\hat{s}\right)}{\xi_{k}/\hat{s}}$$

Myriad weight function is

(18) $$\upsilon_{k}=\frac{\delta^{2}}{\delta^{2}+{\left(\xi_{k}/\hat{s}\right)}^{2}}$$

where $\hat{s}$ is a robust regression of the standard deviation of the LSSVM error variables $\xi_{k}$:

(19) $$\hat{s}=\frac{IQR}{2\times 0.6745}$$

where IQR is the interquartile range, that is, the difference between the two data at 75% and 25%, respectively, after sorting the error values

(20) $$\hat{s}=1.483\times \mathrm{MAD}\left(x_{i}\right)$$

where $\mathrm{MAD}\left(x_{i}\right)$ stands for the median absolute deviation. we use the robust version of cross-validation, three of the four weight functions contain parameters which have to be tuned The cross-validation automatically tunes the parameters of the Huber and myriad weight function according to the best performance for these two weight functions. The two parameters of the Hampel weight function are set to $b_{1}=2.5$ and $b_{2}=3$.

The IR-LSSVM requires to obtain the distribution information of the error based on the training of the LSSVM, and then the weight $\upsilon_{k}\left(k=1,\cdots ,N\right)$ is set to minimize F(x), and finally the weighted LSSVM is trained again [26].

**IR-LSSVM Algorithm** is as follows:

1. Given training data ${\left\{x_{k},y_{k}\right\}}_{k=1}^{N}$, find an optimal $\left(c,\sigma^{2}\right)$ combination (by ten-fold cross-validation or generalization bounds) by solving systems (8).
2. For the optimal $\left(c,\sigma^{2}\right)$ combination one computers $\xi_{k}=\alpha_{k}/c$ from (8).
3. Computer $\hat{s}$ from the $\xi_{k}$ distribution.
4. Determine the weights $\upsilon_{k}$ based on $\xi_{k}$, $\hat{s}$, besides, a suitable weight function is selected from (15) to (18).
5. Solve the weighted LSSVM (14), giving the model $y\left(x\right)=\sum_{k=1}^{N}\alpha_{k}^{*}K\left(x,x_{k}\right)+b^{*}$.

## 3. Experimental Configuration

**Figure 5:**
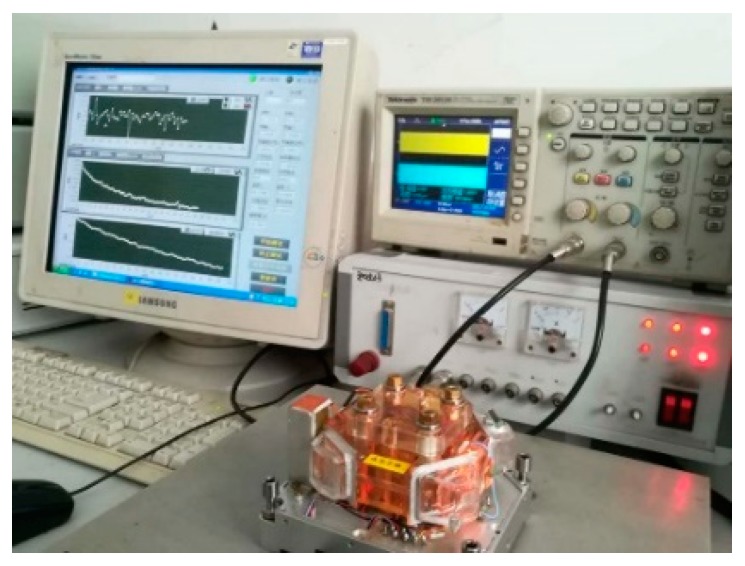
TRPLG used in the temperature experiment.

The gyro used in the temperature experiment was the type 70 TRPLG produced by Xi’an North Jierui Optoelectronics Technology Ltd. (Xi’an, China), and is shown in Figure 5.

The large temperature cycle was set up as follows: (1) the TRPLG was fixed with bolts in the temperature chamber, and tested for 2 h at 25 °C, (2) the temperature was then decreased at the rate of 1 °C/min until −40 °C and tested for 6 h, (3) increased at the rate of 1 °C/min to 70 °C and tested for 10 h, (4) decreased at the rate of 1 °C/min until 25 °C and tested for 4 h. The entire cycle was performed twice, once for training and once for compensation.

**Figure 6:**
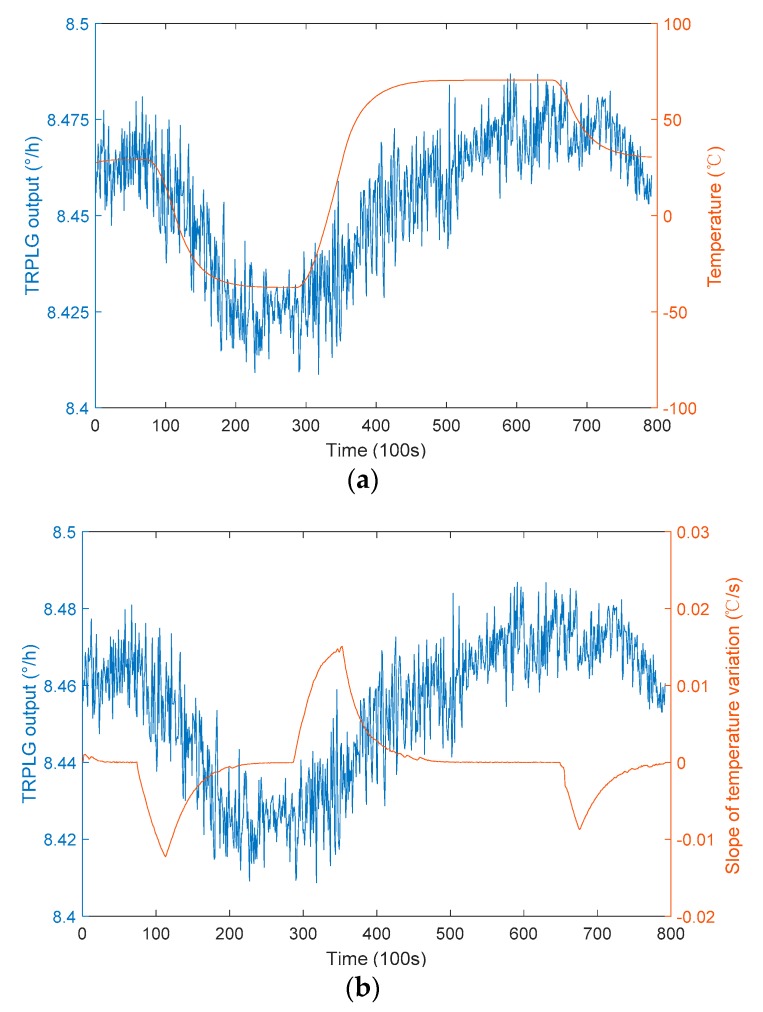

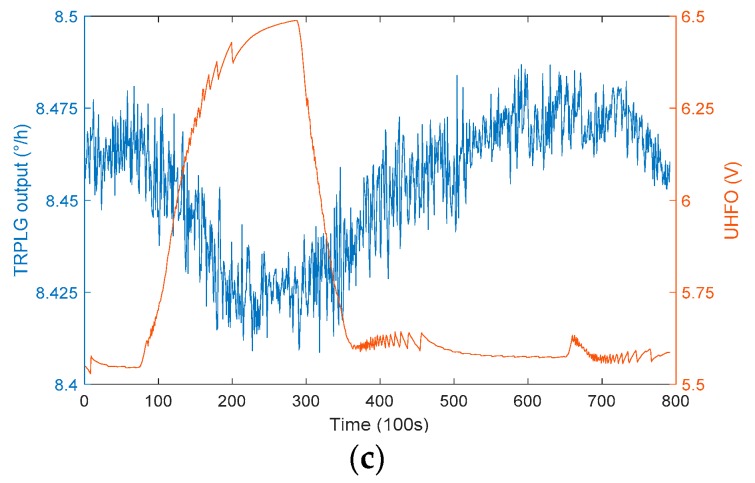
The relationship between the TRPLG output and different parameters: (**a**) Temperature; (**b**) Slope of temperature variation; (**c**) UHFO.

The test software provided the data of temperature, slope of temperature variation, UHFO and TRPLG output, the slope of temperature variation based on the temperature difference per unit time, at intervals of 100 s. The relationship between the TRPLG output and different parameters in the large temperature cycling experiments are shown in Figure 6. TRPLG output exhibited a complex nonlinearity with the slope of temperature variation, which can greatly affect the performance of the TRPLG. The TRPLG output exhibited a good correlation with the temperature. Overall, the UHFO not only correlated well with the TRPLG output but also had better local characteristics. The correlation coefficients of these three parameters to the TRPLG output are shown in Table 1. The correlation between the UHFO and TRPLG output was the strongest, indicating that the TRPLG bias compensation using UHFO would achieve a better result.

## 4. Analysis and Discussion of Results

### 4.1. Bias Compensation Using LS(least squares) Model

For further comparison, the traditional LS model of TRPLG bias is given by [16]

(21) $$B=k_{0}+k_{1}X+k_{2}X^{2}+k_{3}X^{3}$$

where B is TRPLG bias, X is TRPLG parameter, and $k_{i}\left(i=0,1,2,3\right)$ are LS fitting coefficients.

**Figure 7:**
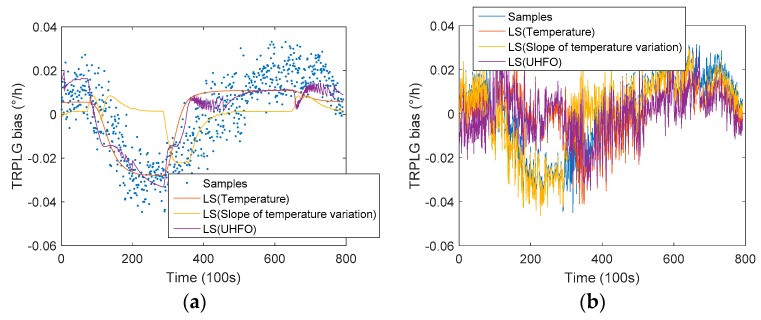
TRPLG bias compensation using different parameters based on LS model: (**a**) Results of regression; (**b**) Results after compensation.

The TRPLG bias can be obtained by subtracting the projection of the earth’s rotation angle rate at this latitude from the TRPLG output. The compensation results of the TRPLG bias using different parameters based LS model are shown in Figure 7. TRPLG bias compensation using slope of temperature variation improved little on the raw data when compared with the model using other two parameters. TRPLG bias compensation using UHFO showed a slightly better result when compared with the model using temperature. Because the LS model was subject to certain restrictions due to complex nonlinear relationships, the compensation result was not satisfactory. Table 2 describes the TRPLG bias stability for original and compensated data based on LS model using different parameters. UHFO improved the TRPLG bias stability by 40.54% to reach the maximum, which was 7.87% higher than traditional temperature compensation.

### 4.2. Bias Compensation Using Stepwise Regression Model

To improve the regression effect, we introduced two multivariate stepwise regression models. Model 1 is a traditional second-order model based on temperature, slope of temperature variation, and its cross terms. The expression is

(22) $$B=k_{0}+k_{1}T+k_{2}T^{2}+k_{3}\frac{dT}{dt}+k_{4}{\left(\frac{dT}{dt}\right)}^{2}+k_{5}T\frac{dT}{dt}$$

where T is the temperature, dT/dt is the slope of temperature variation, $k_{i}\left(i=0,1,\cdots ,5\right)$ are regression coefficients.

This study used interactive stepwise regression analysis to find the optimal regression equation. First, the first three items included in the initial model were specified as T, $T^{2}$, and dT/dt. Then, the upper limit of the significance probability of entering the model was set to 0.05, and the variable with the P value of the significance test less than 0.05 was likely to be introduced into the model. The lower limit of the significance probability of removing the variable from the model was 0.1, and the variable with the value of the significance test greater than 0.1 might be excluded from the model. Finally, the stepwise regression model is

(23) $$\begin{array}{c} B=-1.427\times 10^{-3}+4.906\times 10^{-4}T-4.616\times 10^{-6}T^{2}-0.917\frac{dT}{dt}-57.349{\left(\frac{dT}{dt}\right)}^{2}\\ -1.926\times 10^{-2}T\frac{dT}{dt} \end{array}$$

**Figure 8:**
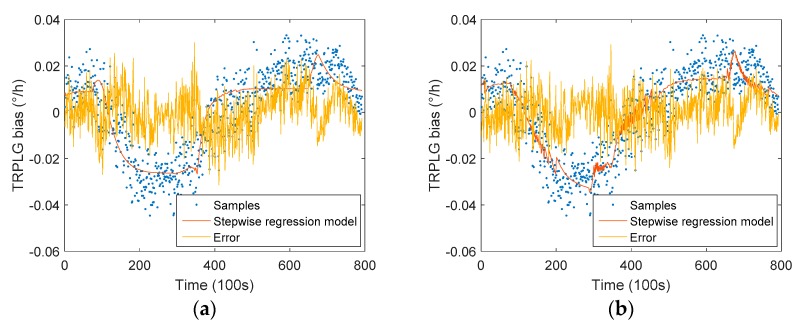
TRPLG bias compensation based on stepwise regression model: (**a**) Results of Model 1; (**b**) Results of Model 2.

The compensation result of Model 1 is shown in Figure 8a. It can be seen that the compensation effect was better than the LS model, and the fluctuation of the middle portion was reduced.

As seen in Table 1, given that the TRPLG output has the strongest correlation and the best LS compensation result with the UHFO, as seen in Table 2, we established a second-order stepwise regression model based on temperature, slope of temperature variation, UHFO, and its cross terms. The expression of Model 2 is

(24) $$B=k_{0}+k_{1}T+k_{2}T^{2}+k_{3}\frac{dT}{dt}+k_{4}{\left(\frac{dT}{dt}\right)}^{2}+k_{5}U+k_{6}U^{2}+k_{7}T\frac{dT}{dt}+k_{8}TU+k_{9}\frac{dT}{dt}U$$

where U is the UHFO, $k_{i}\left(i=0,1,\cdots ,9\right)$ are regression coefficients.

The variables in Model 2 were introduced and removed using the interactive stepwise regression analysis method in Model 1. Ultimately, the ${\left(dT/dt\right)}^{2}$ was eliminated, and the expression of the regression equation is

(25) $$\begin{array}{c} B=-1.565+8.292\times 10^{-3}T+5.529\times 10^{-6}T^{2}+26.263\frac{dT}{dt}+0.646U-6.494\times 10^{-2}U^{2}\\ -4.055\times 10^{-2}T\frac{dT}{dt}-1.562\times 10^{-3}TU-4.666\frac{dT}{dt}U \end{array}$$

The compensation result of Model 2 is shown in Figure 8b. Model 2 reduced overall fluctuations when compared with Model 1. Additionally, Model 2 improved the TRPLG bias stability by 54.49%, which was 10.62% higher than Model 1, as seen in Table 3. The introduction of the UHFO improved the TRPLG bias stability by 10.62% when compared with the traditional stepwise regression model.

### 4.3. Bias Compensation Using IR-LSSVM Model

**Figure 9:**
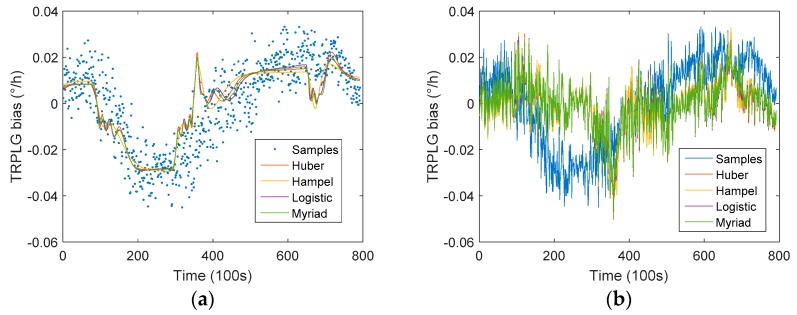

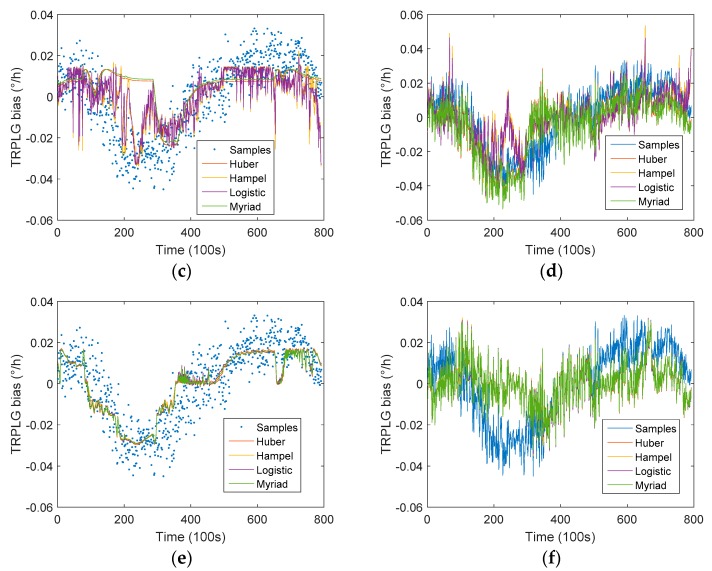
TRPLG bias compensation using different weight functions based on IR-LSSVM model: (**a**) Regression based on temperature; (**b**) Compensation based on temperature; (**c**) Regression based on slope of temperature variation; (**d**) Compensation based on slope of temperature variation; (**e**) Regression based on UHFO; (**f**) Compensation based on UHFO.

The IR-LSSVM algorithm is shown in Section 2.3. We used four different weight functions and we used different single parameters as the input and the TRPLG bias as the output to establish an IR-LSSVM model. The regression and compensation results are shown in Figure 9, which shows that the IR-LSSVM model of TRPLG bias compensation using UHFO was better than that using other two parameters. In comparison to the LS model, the IR-LSSVM model achieved better compensation results.

Table 4 describes the TRPLG bias stability for compensated data based on IR-LSSVM model using different single parameters. IR-LSSVM based on Humber improved the TRPLG bias stability by 45.46% to reach the maximum; moreover, the increased percentage using UHFO as an input parameter is higher than the others overall. Therefore, the importance of the UHFO parameters was verified again.

**Figure 10:**
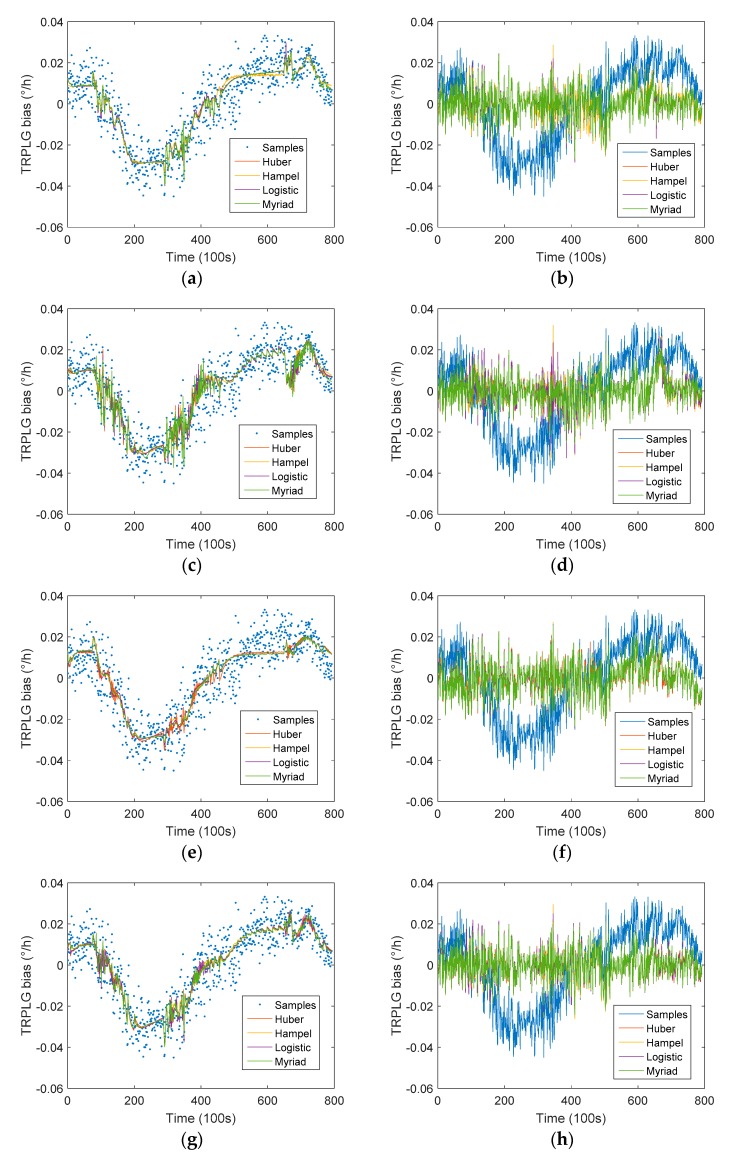
TRPLG bias compensation using different weight functions and composite parameters based on IR-LSSVM model: (**a**) Regression based on temperature and slope of temperature variation; (**b**) Compensation based on temperature and slope of temperature variation; (**c**) Regression based on temperature and UHFO; (**d**) Compensation based on temperature and UHFO; (**e**) Regression based on slope of temperature variation and UHFO; (**f**) Compensation based on slope of temperature variation and UHFO; (**g**) Regression based on all parameters; (**h**) Compensation regression based on all parameters.

The IR-LSSVM model of TRPLG bias compensation using single parameter cannot completely eliminate TRPLG bias drift. To complete high precision compensation, a bias composite compensation method based on IR-LSSVM model was used. We selected different composite parameters as inputs and TRPLG bias as outputs; the regression and compensation results are shown in Figure 10. When two parameters were selected as inputs, most of the TRPLG bias drift was eliminated, with only local drift at the end of the data. When all parameters were selected as inputs, TRPLG bias drift was basically eliminated.

Table 5 describes the TRPLG bias stability for compensated data based on IR-LSSVM model using different composite parameters. When all parameters were selected as inputs, the myriad weight function improved the TRPLG bias stability by 61.19% to reach the maximum. The compensation effect was better than the stepwise regression method.

To clearly illustrate the significance of the UHFO, we compared Table 4 and Table 5. When temperature and UHFO were used as composite parameters, the myriad weight function improved the TRPLG bias stability by 59.84%. It was 31.77% better than using temperature as the sole input. When slope of temperature variation and UHFO were used as inputs as composite parameters, the Huber weight function improved the TRPLG bias stability by 55.78%, which was 39.68% better than only using slope of temperature variation as an input. In addition, when all parameters were selected as inputs, TRPLG bias stability was 4.52% higher than using temperature and slope of temperature variation as inputs.

In summary, the UHFO is the most important parameter in the process of TRPLG bias compensation. It can make up for the shortcomings of traditional temperature compensation and can greatly improve the TRPLG bias stability.

We compared the compensation results of IR-LSSVM model and LSSVM model. The LSSVM compensation model is expressed in Equation (10). To obtain an LSSVM model with the RBF kernel, two extra tuning parameters are needed: the regularization parameter c, which determines the trade-off between the training error minimization and smoothness of the estimated function, and the kernel function parameter $\sigma^{2}$. In this study, we used the leave-one-out method to determine the tuning parameters to train the first set of sampling data and produced the LSSVM model for the TRPLG bias compensation.

**Figure 11:**
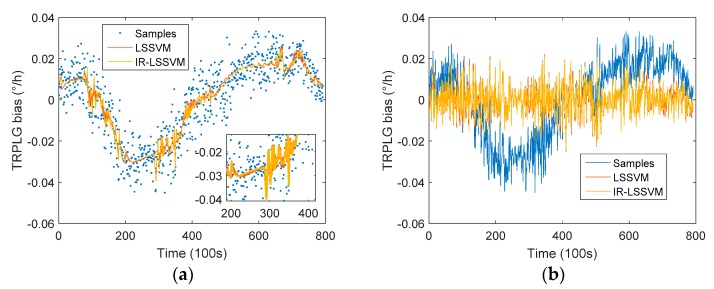
TRPLG bias compensation based on LSSVM model and IR-LSSVM model: (**a**) Regression based on all parameters; (**b**) Compensation based on all parameters.

The comparison of compensation results between the IR-LSSVM model and the LSSVM model is shown in Figure 11. With the LSSVM model, the TRPLG bias stability was improved by 59.03%, which was smaller than the compensation effect (61.19%) of the IR-LSSVM model. Therefore, the IR-LSSVM model that considered the UHFO parameter realized high precision compensation for TRPLG bias.

## 5. Conclusions

To eliminate the complex TRPLG bias variation and improve TRPLG bias stability, we theoretically analyzed the factors affecting the TRPLG bias. The UHFO was introduced into TRPLG bias compensation based on temperature and slope of temperature variation; comparative analysis was performed in LS regression, stepwise regression, and IR-LSSVM. Large temperature cycling experiments can draw the following conclusions.

First, in comparison with LS regression, multivariate stepwise regression can reduce partial TRPLG bias drift.

Second, IR-LSSVM based on composite parameters can overcome the shortcomings of poor nonlinear fitting ability of the LS regression and stepwise regression.

Third, the UHFO proved to be the most important parameter for TRPLG bias compensation; it can make up for the shortcoming of traditional compensation based on temperature-related parameters and can greatly improve the TRPLG bias stability.

Fourth, when temperature, slope of temperature variation, and UHFO are used as inputs, the IR-LSSVM based on myriad weight function can completely eliminated TRPLG bias drift to achieve high precision compensation.

In conclusion, the IR-LSSVM model proves to be accurate, reliable, and has a significant practical value in engineering.

## Figures and Tables

**Table 1:** Correlation coefficient between TRPLG output and parameters.

	Temperature	Slope of Temperature Variation	UHFO
Correlation coefficient with TRPLG output	0.71	−0.43	−0.82

**Table 2:** TRPLG bias stability for compensated data based on LS model using different parameters.

		Parameters Based on LS Model		
		Temperature	Slope of Temperature Variation	UHFO
TRPLG bias stability (°/h)	Before compensation	0.01850	0.01850	0.01850
	After compensation	0.01194	0.01702	0.01100
Improvement		35.46%	8.00%	40.54%

**Table 3:** TRPLG bias stability for compensated data based on two stepwise regression models.

		Model 1	Model 2
TRPLG bias stability (°/h)	Before compensation	0.01850	0.01850
	After compensation	0.00942	0.00842
Improvement		49.08%	54.49%

**Table 4:** TRPLG bias stability for compensated data based on IR-LSSVM model using different single parameters.

No.	Parameters	Weight Function	c	$\sigma^{2}$	F(x) $\left(\times 10^{-3}\right)$	Bias Stability (°/h)	Improvement
1	T	Huber	$1.5958\times 10^{2}$	0.0356	8.5410	0.01105	40.27%
		Hampel	78.6103	0.4086	8.5280	0.01123	39.30%
		Logistic	$9.3665\times 10^{8}$	0.2138	8.4047	0.01089	41.14%
		Myriad	$1.9285\times 10^{4}$	0.0742	8.4990	0.01090	41.08%
2	dT/dt	Huber	$8.2961\times 10^{3}$	7.2794	13.1121	0.01712	7.46%
		Hampel	1.0207	0.0003	12.6074	0.01357	26.65%
		Logistic	1.4421	0.0003	12.7182	0.01356	26.70%
		Myriad	1.0537	3.8071	13.1177	0.01714	7.35%
3	U	Huber	0.2718	0.0044	8.1178	0.01009	45.46%
		Hampel	0.1643	0.0065	8.1610	0.01024	44.65%
		Logistic	0.2691	0.0070	8.1394	0.01021	44.81%
		Myriad	0.1955	0.0060	8.1437	0.01015	45.14%

**Table 5:** TRPLG bias stability for compensated data based on IR-LSSVM model using different composite parameters.

No.	Parameters	Weight Function	c	$\sigma^{2}$	F(x) $\left(\times 10^{-3}\right)$	Bias Stability (°/h)	Improvement
1	T,dT/dt	Huber	$1.2538\times 10^{3}$	0.2065	6.5456	0.00755	59.19%
		Hampel	1.1991	0.0930	6.6019	0.00794	57.08%
		Logistic	$3.5476\times 10^{2}$	0.1399	6.5949	0.00757	59.08%
		Myriad	$1.8752\times 10^{3}$	0.2041	6.5080	0.00752	59.35%
2	T,U	Huber	$4.3846\times 10^{3}$	0.0390	7.0111	0.00789	57.35%
		Hampel	79.8662	0.0297	7.1130	0.00823	55.51%
		Logistic	$6.2443\times 10^{2}$	0.0304	7.0946	0.00807	56.38%
		Myriad	8.4177	0.0045	7.1402	0.00743	59.84%
3	dT/dt,U	Huber	$2.4444\times 10^{4}$	1.6025	6.7729	0.00818	55.78%
		Hampel	0.7546	0.2386	6.8129	0.00837	54.76%
		Logistic	1.9995	0.2144	6.8975	0.00832	55.03%
		Myriad	1.4267	0.3799	6.9093	0.00838	54.70%
4	T,dT/dt,U	Huber	$4.0580\times 10^{5}$	2.2799	6.4217	0.00740	60.00%
		Hampel	26.3212	0.1263	6.4479	0.00733	60.38%
		Logistic	$3.4780\times 10^{4}$	1.0623	6.4218	0.00740	60.00%
		Myriad	76.0796	0.1458	6.3711	0.00718	61.19%
		Unweighted	$1.3624\times 10^{3}$	0.9771	6.6791	0.00758	59.03%
